# Aidi Injection as Adjuvant Drug Combined with Chemotherapy in Treatment of Breast Cancer: A Systematic Meta-Analysis

**DOI:** 10.1155/2021/8832913

**Published:** 2021-01-08

**Authors:** Chenhao Wu, Yongjun Qi, Juan Zhou, Chen Yao, Min Miao, Chen Cheng

**Affiliations:** ^1^Breast Surgery Department, Hainan Women and Children's Medical Center, No. 75 Longkun Nan Road, Hai Kou, Hainan Province, China; ^2^Obstetrics and Gynecology Department, Jiangdu People's Hospital of Yangzhou, No. 9 Dongfang Hong Road, Jiangdu District, Yangzhou, Jiangsu Province, China

## Abstract

**Objective:**

To compare the efficacy and safety of combination of Aidi injection and chemotherapy and chemotherapy alone in treatment of breast cancer.

**Methods:**

The related control and randomized studies till August 1^st^, 2020, were retrieved in the database including PubMed, Embase, Cochrane Library, CNKI, CBM, Wang-Fang, and VIP. Primary outcomes were response rate (RR) and performance status (KPS) improvement rate; secondary outcomes were rate of adverse drug reactions (ADR) including myelosuppression, digestive tract reaction, liver dysfunction, and cardiac toxicity. Review Manager 5.3 was used in the present analysis.

**Results:**

In total, 20 studies (18 articles) were included in the present analysis. RR (OR 1.76 (1.32, 2.35); *p*=0.0001) and KPS improvement rate (OR: 2.68 (1.34, 6.46); *p*=0.007) in Aidi injection plus chemotherapy group were significantly higher than those of chemotherapy alone group. Addition of Aidi injection significantly reduced the rate of myelosuppression, digestive tract reaction, leukocyte decrease, II-IV cardiac function abnormality, atrial dysrhythmia, ventricular arrhythmia, ST segment T wave inversion, and abnormal ECG (all *p* < 0.05).

**Conclusion:**

Aidi injection could increase the efficacy of chemotherapy, could reduce myelosuppression, digestive tract reaction, and cardiac toxicity induced by chemotherapy, and did not lead to additional toxicity and side effect. Therefore, it is an anticancer drug with good efficacy and low toxicity, worth further popularization.

## 1. Introduction

Breast cancer is a common cancer type and is the fifth most common cause of cancer death [1]. In 2012, there were 1.67 million newly diagnosed breast cancer cases, comprising 25% of all sorts of cancers among females [2]. In China, the number of breast cancer cases increased dramatically. The number of newly diagnosed breast cancer cases in 2000 was 121,2693, and this number reached 168,013 in 2005 [3], and 278900 in 2014 [4]. The main options in breast cancer treatment included surgery, chemotherapy, and radiation. Among them, chemotherapy has been a widely accepted and applied treatment method. However, chemotherapy usually temporarily relieves symptoms, lengthens survival, but occasionally cures the disease, so its treatment efficacy is still to be improved [5]. On the other hand, the related toxicity greatly affected quality of life of patients. Therefore, much work has been made to find alternative treatment methods. In recent years, Traditional Chinese Medicine (TCM) received much attention in the field of cancer treatment due to its capability of efficacy improvement and toxicity reduction [6].

Aidi injection was one of the Chinese Patent Medicines with anticancer acitivity included in Catalogue of Drugs for Basic National Medical Insurance and Countermeasures of China [7]. It is extracted from Chinese herbal medicines of catharides, ginseng, astragalus, and acanthopanax senticosus. In opinion of TCM, Aidi injection has the ability of clearing away heat and toxin, removing stasis, and dispersing accumulation. Cantharidin is the major component for toxicity of cantharides and also is an effective anticancer component. It can inhibit cancer cells without decreasing the pericirculation leucocytes level and has obvious immune-suppression effect, which is quite outstanding for current anticancer drugs [8]. Ginseng, astragalus, and acanthopanax senticosus have showed anticancer capability of inducing apoptosis of cancer cells, inhibiting proliferation of cancer cells, inhibiting cancerous angiogenesis, and so on, and they usually also have features of low toxicity, low drug resistance, and obvious immune-improvement capability. In China, Aidi injection was extensively used in the treatment of primary liver cancer [9], lung cancer [10], gastric cancer [11], colon cancer [12], lymphadenoma [13], gynecology cancer [14], and so on. In clinical practice, Aidi injection is usually used as adjuvant drug in chemotherapy in cancer treatment to decrease toxicity of chemotherapy and improve quality of life [15]. There have been a number of clinical studies investigating combination of Aidi injection with chemotherapy in cancer treatment. In the studies among breast cancer patients, addition of Aidi injection had showed certain benefits. Therefore, we performed this meta-analysis to systematically review and evaluate the efficacy and safety of Aidi injection as adjuvant drug in chemotherapy in the treatment of breast cancer.

## 2. Materials and Methods

### 2.1. Inclusion and Exclusion Criteria

The inclusion criteria were as follows: (1) types of studies: randomized controlled trials; retrospective or observational studies were not eligible; (2) types of participants: pathologically or cytologically diagnosed with breast cancer; Karnofsky performance status score ≥60; (3) intervention: patients in control group received pure chemotherapy and the patients in the experimental group received chemotherapy plus Aidi injection; (4) outcome measurements: at least one of the three measurements was evaluated: short-term response rate for solid tumor, performance status improvement rate, and adverse events rate.

Exclusion criteria were as follows: (1) other Traditional Chinese Medicines were used during study period; (2) other cancer treatments such as radiation therapy or immunological or target treatment were used during study period.

### 2.2. Retrieval Strategy

We searched the related studies till August 1^st^, 2020, using the database including PubMed, Cochrane library, Embase, and Chinese medical databases: CNKI, CBM, Wang-Fang, and VIP. The retrieval terms were “Aidi injection” or “Aidi”, AND “breast cancer”, OR “breast tumor” OR “breast carcinoma” in retrieval using PubMed, Cochrane library, and Embase. These terms in English and Chinese both were used for CNKI, CBM, Wang-Fang, and VIP. The references of important articles were also searched.

### 2.3. Literature Screening, Data Extraction, and Quality Assessment

Two independent reviewers (WCH and QY) made the literature screening and data extraction, respectively. For each literature screening, duplication, title, abstract, and main text were examined. The screening results by the two reviewers were compared and decided through discussion. When agreement could not be made with discussion, a third independent reviewer (YC) was invited to make final decision. The extracted information included author name, publication year, sample size, TNM stage, intervention methods, treatment cycles, and outcome measurements.

Quality assessments were made with Cochrane risk of bias tool as suggested by Cochrane handbook for systematic reviews of interventions. The assessed measurements include random sequence generation, allocation concealment, blinding of participants and personnel, blinding of outcome assessment, incomplete outcome data, and selective reporting. Each measurement was rated with low, unclear, and high bias risk.

### 2.4. Outcome Measurements

Short-term treatment efficacy was determined according to the modified Response Evaluation Criteria in Solid Tumours 1.1 (RECIST 1.1) [16]. Response rate = number of patients with complete response + number of patients with partial response rate/total patient number. Performance status was assessed with Karnofsky Performance Status (KPS) score. After treatment, increase of KPS score from baseline ≥10 was considered as KPS improvement; decrease of KPS score from baseline ≥10 was considered as KPS reduction; KPS score above or below <10 was considered as stable KPS. KPS improvement rate = number of patients with KPS improvement/total patient number. The toxicity and adverse events were assessed as I°, II°, III°, and IV°, according to the common toxicity criteria of chemotherapy drugs drafted by WHO (1991); cardiac function abnormality was also assessed according to the WHO anticancer drug toxicity criteria. Grade 0 indicates that heart rate, rhythm, and function are normal; I°indicates no symptoms of cardiac insufficiency and may present abnormal cardiac signs; grade II° indicates transient cardiac insufficiency, no treatment needed; grade III° indicates presenting symptoms of cardiac function insufficiency which are manageable; IV° indicates presenting symptoms of cardiac function insufficiency which are unmanageable.

### 2.5. Statistical Analysis

Review Manager 5.3 was used for meta-analysis. Odds ratios (OR) for categorial or continuous variables of experimental group (Aidi injection plus chemotherapy) and control group (chemotherapy alone) were calculated and compared. *P* < 0.05 was considered to be statistically significant. The heterogeneity between the included studies was tested with the standard Chi-squared (*I*^2^Q) test. If *p* < 0.1, *I*^2^ <50%, fixed effect model was used for analysis; if *p* < 0.1, *I*^2^ >50%, random effect model was used. Subgroup analyses according to number of cycles (≤3 vs >3) were made to identify the heterogeneity source. Funnel plot was used for estimation of publication bias. If the number of included studies was less than 10, publication bias was not assessed.

## 3. Results

### 3.1. Literature Screening Results and Characteristics of the Included Studies

**Figure 1:**
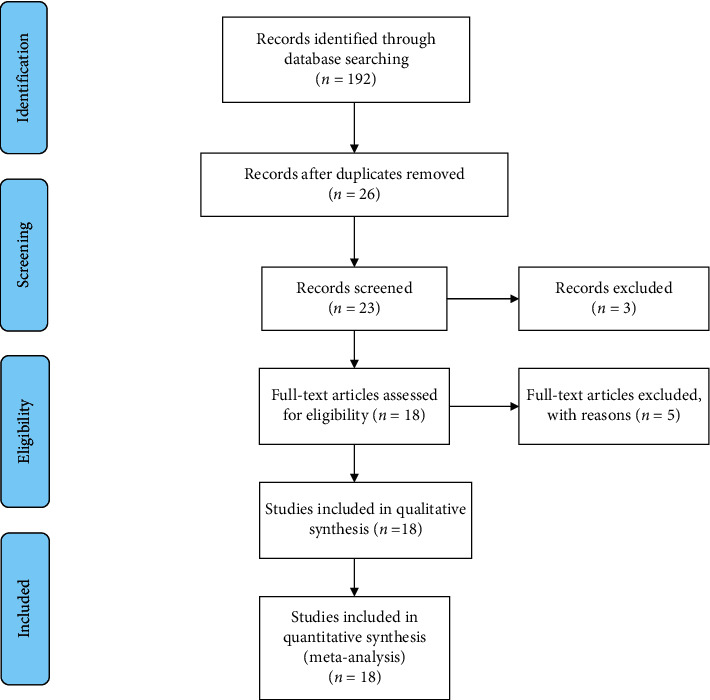
Flowchart of the studies selection process.

Following the search strategy described above, 96 articles were retrieved. Among them, 23 were retrieved from CNKI, 26 were retrieved from CBM, 22 were retrieved from VIP, 24 were retrieved from Wan-Fang, 1 was retrieved from PubMed, 0 were retrieved from EMBASE, and 0 were retrieved from Cochrane library. After duplicate checking, 26 articles were obtained. After title and abstract examination, 3 articles were excluded. After main-text examination, 5 articles were excluded: 1 study did not evaluate the measurements of response rate or performance status improvement rate; 1 study did not provide TNM stage of included patients; 1 study was single arm study rather than randomized controlled study; 1 study was performed among patients with cancer other than breast cancer; 1 study used TCMs other than Aidi injection. Finally, 18 articles (20 studies) were included in the present meta-analysis. The flowchart of article selection process is shown as Figure 1.

The characteristics of the 20 included studies are shown in Table 1 [17–34]. All the 20 studies were published in Chinese journals. The study performed by Gao et al. compared Aidi plus chemotherapy and chemotherapy alone, respectively, among treatment naïve patients and patients with drug resistance for anthracycline and paclitaxel, so the two comparisons were both included and regarded as two studies: Gao QH 2013a and Gao QH 2013b [21]. The study performed by Wang GD et al. compared low-dose Aidi plus chemotherapy and high-dose Aidi plus chemotherapy and chemotherapy alone, so two comparisons (low dose vs control; high dose vs control) were included and regarded as two studies: Wang GD 2012a and Wang GD 2012b [30]. The patients in four studies performed by Chen WM et al. [17], Han et al. [22], Wang GD et al. [30], and Jin et al. [23] received chemotherapy after surgery. The other studies out of the four studies did not administrate surgery. The chemotherapies were regular regimen but varied among the studies. The dosage of Aidi injection ranged from 50 ml to 100 ml.

### 3.2. Quality Assessment Result

**Figure 2:**
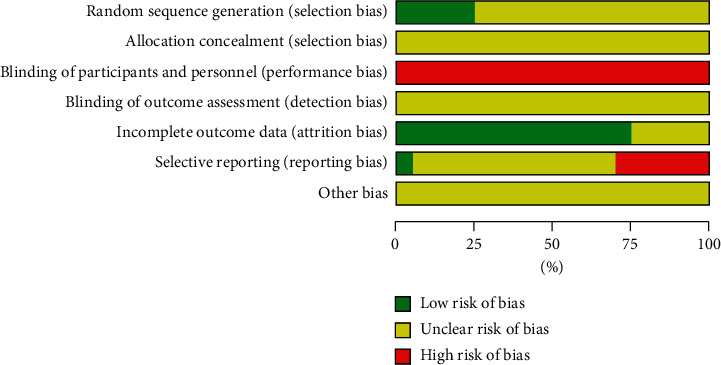
Risk of bias graph of included studies.

**Figure 3:**
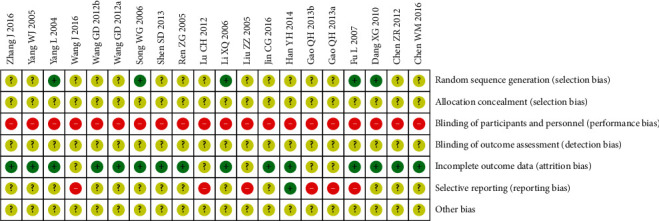
Summary of risk bias of included studies.

All the included studies were randomized controlled studies. The baseline characteristics of patients were well balanced between the experimental group and control group. Five studies reported the randomized sequence generation methods [19, 20, 25, 29, 32]; 5 studies had evaluated long-term efficacy so they reported follow-up [20, 27, 29, 31, 34] and the rest studies did not as they only evaluated short-term efficacy; 6 studies evaluated adverse events rate and reported multiple adverse events but did not provide overall data of all adverse events that occurred [20, 21, 24, 26, 31] and the rest of studies had made complete reports of all or certain adverse events; 1 study reported drop-out rate [22]. However, in all the studies, the blindness of allocation and assessment was not described, so they were rated as unclear; the blindness of participants and personals was not described either and was rated as high risk based on the general practice in hospital of China (Figures 2 and 3).

### 3.3. Outcomes

#### 3.3.1. Response Rate

**Figure 4:**
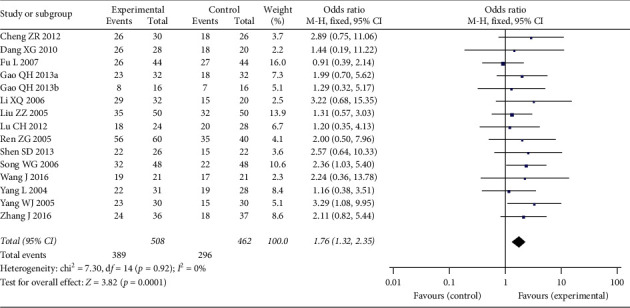
Forest plot of response rate in breast cancer patients receiving chemotherapy alone and chemotherapy plus Aidi injection.

**Figure 5:**
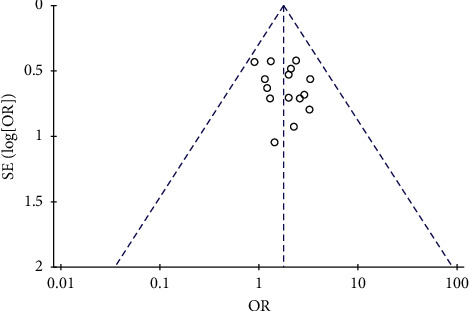
Funnel plot of response rate for the publication bias.

15 studies were included in the analysis of response rate, which included 970 patients, 508 in Aidi plus chemotherapy group and 462 in chemotherapy alone group (Figure 4). The heterogeneity between the included studies was not significant (*p*=0.92; *I*^2^ = 0%), so fixed-effect model was used. The analysis results indicated that the pooled response rate in Aidi plus chemotherapy group was significantly higher than that of the chemotherapy alone group (OR 1.76 (1.32, 2.35); *p*=0.0001). Funnel plot was adopted to estimate the publication bias and the result suggested no obvious publication bias (Figure 5).

#### 3.3.2. Performance Status Improvement Rate

**Figure 6:**
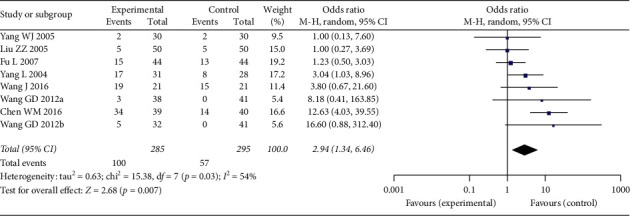
Forest plot of performance status improvement rate in patients receiving chemotherapy alone and chemotherapy plus Aidi injection.

Eight studies were included in the analysis of performance status improvement rate, which included 700 patients, 345 in Aidi plus chemotherapy group and 355 in chemotherapy alone group (Figure 6). Heterogeneity between the included studies was significant (*p*=0.03, *I*^2^ = 54%), so random-effects model was used. The analysis result showed that performance status improvement rate in the patients receiving Aidi plus chemotherapy was significantly higher than that in the patients receiving chemotherapy alone (OR: 2.68 (1.34, 6.46); *p*=0.007).

**Figure 7:**
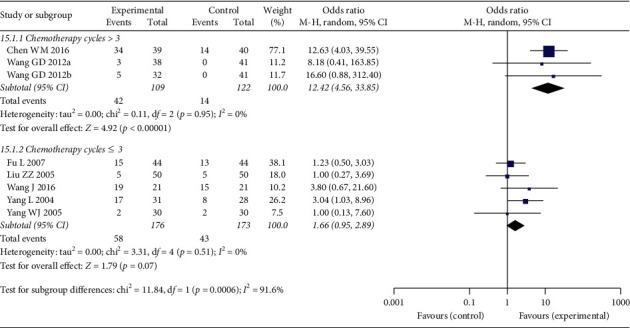
Forest plot of performance status in subgroup analysis regarding chemotherapy cycles.

However, in subgroup analysis in respect to cycle numbers, the difference of performance status improvement rate was significant in the subgroup in which chemotherapy cycles were >3 (OR: 12.42 (4.56, 33.85); *p* < 0.00001), and heterogeneity was not significant (*p*=0.95, *I*^2^ = 0%) (Figure 7); the performance status improvement rate was not significantly different in the subgroup in which chemotherapy cycles were ≦3 (OR: 1.66 (0.95, 2.89); *p*=0.07), and the heterogeneity was not significant between the studies (*p*=0.51, *I*^2^ = 0%) (Figure 7). Furthermore, the heterogeneity between the two subgroups was significant (*p*=0.0006; *I*^2^ = 91.6%) (Figure 7), indicating that chemotherapy cycles >3 or ≤3 were a source of heterogeneity in the total analysis.

**Figure 8:**
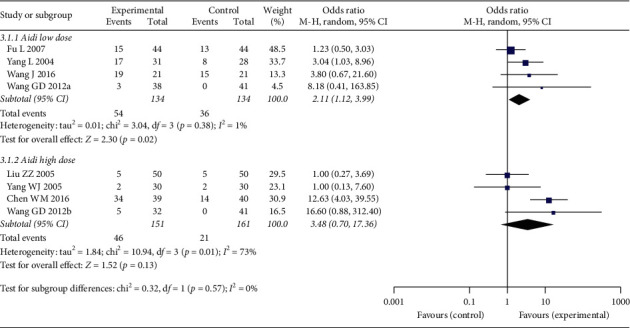
Forest plot of performance status in subgroup analysis regarding Aidi injection high or low dose.

Again, subgroup analysis regarding Aidi injection of high (100 mL) or low (50 mL) dose was also performed (Figure 8) to determine the source of heterogeneity. In the subgroup of low-dose Aidi, heterogeneity was not significant (*p*=0.38, *I*^2^ = 1%). The pooled performance improvement rate was significantly higher in low-dose Aidi plus chemotherapy group [OR: 2.11 (1.12, 3.99); *p*=0.02]. In the subgroup of high-dose Aidi, the heterogeneity between studies was significant (*p*=0.01, *I*^2^ = 73%); the pooled performance improvement rate was not significantly different in this subgroup analysis [OR: 3.48 (0.70, 17.36); *p*=0.13] (Figure 8). However, the difference between the subgroups was not significant (*p*=0.57, *I*^2^ = 0%).

### 3.4. Adverse Drug Events Rate

**Figure 9:**
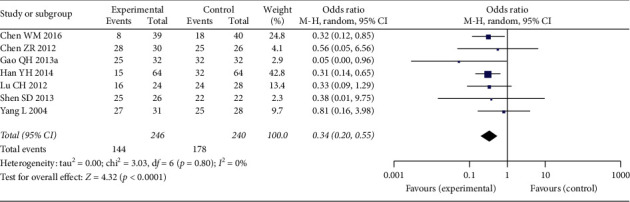
Forest plot of myelosuppression rate among patients receiving chemotherapy alone and chemotherapy plus Aidi injection.

*Myelosuppression Rate*. Seven studies were included in the analysis for myelosuppression rate, which included 486 patients, 246 in Aidi injection plus chemotherapy group and 240 in chemotherapy group (Figure 9). The heterogeneity between the included studies was not significant (*p*=0.8, *I*^2^ = 0%). The analysis result indicated that the pooled myelosuppression rate of Aidi plus chemotherapy group was significantly lower than that of the chemotherapy alone group (OR: 0.34 (0.20, 0.55); *p* < 0.0001).

Furthermore, 4 studies were included in the analysis for III-IV grade myelosuppression rate. Heterogeneity was not significant (*p*=0.25%, *I*^2^ = 28%) (Table 2). The pooled III-IV grade myelosuppression rate was significantly lower in Aidi plus chemotherapy group (OR: 0.43 (0.25, 0.76); *p*=0.03) (Table 2).

**Figure 10:**
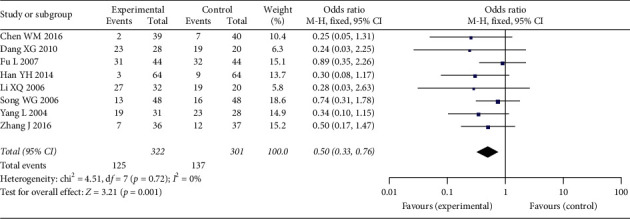
Forest plot of digestive tract reaction rate among patients.

*Digestive Tract Reaction Rate*. Eight studies were included in the analysis for digestive tract reaction rate, which included 623 patients (Figure 10). The heterogeneity between studies was not significant (*p*=0.72, *I*^2^ = 0%). The analysis result indicated that the pooled digestive tract reaction rate was significantly lower in Aidi plus chemotherapy group, when compared with chemotherapy alone group (OR: 0.50 (0.17, 1.47); *p*=0.001).

The analysis for III-IV grade digestive tract reaction rate included 4 studies (Table 2). The heterogeneity was shown between studies (*p*=0.06; *I*^2^ = 59%). The III-IV grade digestive tract reaction rate was significantly lower in Aidi plus chemotherapy group (OR: 0.42 (0.22, 0.80); *p*=0.008).

*Other ADRs*. The other adverse drug reaction rates were also compared, as shown in Table 2. The pooled total and II-IV grade leukocyte decrease rate were shown to be significantly lower in Aidi plus chemotherapy group (both *p* < 0.05). The adverse events rates related to cardiac toxicity, including II-IV cardiac function abnormality, atrial dysrhythmia, ventricular arrhythmia, ST segment T wave inversion, and abnormal ECG, were significantly lower in Aidi plus chemotherapy group (all *p* < 0.05). The total and III-IV grade liver dysfunction rate, total and III-IV hair loss rate, phlebitis rate, and atrioventricular block rate were not significantly different between the two groups (all *p* < 0.05).

## 4. Discussion

Side effects in the treatment of breast cancer seriously affect the quality of life of patients and make development of alternative treatment necessary. In hospital of China, Chinese Patent Medicine Aidi injection was widely used as adjuvant drug in chemotherapy for breast cancer. We performed this meta-analysis to compare the efficacy and safety of Aidi injection plus chemotherapy and chemotherapy alone in treatment of breast cancer.

Our analysis demonstrated that addition of Aidi to chemotherapy significantly improved response rate and performance status improvement rate; Aidi plus chemotherapy group significantly decreased the rate of all grade and III-IV grade myelosuppression, the rate of all grade and III-IV grade digestive tract reaction, the rate of all grade and II-IV grade leukocyte decrease, and the rate of most of cardiac toxicity related adverse events. In regards of the other adverse drug reactions, the ADR rate was slightly reduced in Aidi plus chemotherapy group but showed no significance.

The heterogeneity in the analysis for response rate, myelosuppression rate, and digestive tract reaction rate was not significant but was significant in the analysis for performance status improvement rate. In order to identify source of heterogeneity, we made subgroup analysis according to chemotherapy cycle number and Aidi of high or low dosage, respectively. The subgroup analysis demonstrated that the chemotherapy cycle number, namely, the treatment duration of Aidi, was a source of heterogeneity, since the difference between two subgroup analyses was significant. We speculate that TCM usually takes a longer action time; on the other hand, longer Aidi usage may increase its effect compared with shorter usage. The two aspects together might explain the increased KPS improvement rate in subgroup with chemotherapy cycle number ≥3.

The high dose Aidi might increase the KPS improvement rate since OR in high-dose subgroup analysis was higher than OR in low-dose subgroup analysis, but the difference between the high-dose and low-dose subgroup was not significant. Therefore, the dosage of Aidi might not be the source of heterogeneity in the total analysis for KPS improvement rate. We speculate that the high-dose and low-dose Aidi might not be very different regarding their effect on KPS, but it is also possible that the sample size is not large enough to identify their difference.

Chemotherapy is important treatment option for breast cancer for its clinical benefit, but it can also lead to side effects such as myelosuppression, digestive tract reactions, and cardiac toxicity. Its toxicity apparently affects patients that sometimes patients could not complete the whole chemotherapy cycle number. Our study demonstrated that addition of Aidi injection could significantly reduce the chemotherapy related toxicities, and Aidi injection itself did not show any obvious toxicity. The safe profile of Aidi injection had been validated by previous studies; for example, in the study by Hu et al., among the cancer patients who received Aidi injection in 2013–2015 in their department, the adverse drug reaction rate was about 1% [35]. Thus, we may conclude that Aidi injection has obvious toxicity-protection effect during chemotherapy treatment, which might partially explain the improved quality of life of patients, and the increased response rate since it may help patients to better complete chemotherapy courses.

Many studies explored the mechanism for activity of Aidi injection in cancer treatment. Aidi injection is prepared from Cantharidin, Ginsen, Astragaloside, and Acanthopanax senticosus. These Chinese herbal medicines have the ability to inhibit cancers and improve immunity as evidenced by a series of laboratory studies [36–39]. Cantharidin, as the central component in the whole preparation, is featured with anticancer activity and no concurrent myelosuppression [40, 41]. Experimental studies reported that cantharidin sodium injection is effective in the management of breast cancer [42], through inhibiting proliferation of breast cancer cells [43], suppressing authophage, and inducing apoptosis [44]. Aidi injection as the whole preparation has shown anticancer activity and ability of immunological function improvement [45]. For laboratory studies, it was reported that addition of Aidi injection to chemotherapy could significantly increase tumor inhibition rate and reduce Her-2/neu expression level in the nude mice breast carcinoma implanted of Her-2/neu over expression [46]; it could significantly inhibit cancer cell proliferation, induce cell apoptosis, and reduce cell diameter in the ErbB2 positive breast cancer cell BT-474, SK-BR-3, and HCC-1954 [47]; it significantly inhibited proliferation of MCF-9 cells in dose-dependent manner, accompanying altered expression profiles of microRNAs in MCF-9 cells [48]. For human studies, it was reported that Aidi injection treatment could reverse the suppression or promotion effect of chemotherapy on the peripheral blood level of CD3+, CD4+, CD8+, CD4+/CD8+, and NK cells [17, 22, 24, 26, 32, 33, 49]; Aidi injection plus chemotherapy decreased level of tumor markers CEA, CA153 more than chemotherapy alone [17]; addition of Aidi injection to chemotherapy could reduce serum VEGF level of breast cancer patients, that its anticancer action may be achieved through inhibition of tumor angiogenesis to inhibit proliferation, invasion, and metastasis of tumor cells [50].

*Limitations*. (1) Long-term efficacy is important measurement in breast cancer treatment, but the present study only evaluated short-term efficacy. The long-term efficacy measurements such as overall survival and 5-year survival rate were reported in a few included studies, but the measurements were different so pooled effect size could not be calculated. (2) Some included studies also evaluated the effect of addition of Aidi injection on the level of cardiac enzymes, liver function measurements, immune cells, and tumor markers, but those measurements could not be pooled in meta-analysis due to the presentation way of the results. (3) All studies were performed in China and published in Chinese journals, which may lead to certain bias. (4) The overall quality of the included studies was not high, and the blindness was not described by all the studies, so the results still shall be interpreted cautiously. (5) The adverse events were selectively reported in some included studies: for example, in some studies, multiple adverse events were recorded, but not each event was reported in standard with severity grades that the less common adverse events were roughly described with text, which may lead to reporting bias.

Finally, this is the second meta-analysis evaluated efficacy and safety of Aidi injection plus chemotherapy versus chemotherapy alone among breast cancer patients. Compared with the first published one, our analysis has included larger number of studies, thoroughly discussing adverse events of Aidi injections, and made subgroup analysis to identify the effect of treatment duration and dosage on KPS. According to our analysis, Aidi injection could increase the efficacy of chemotherapy and reduce myelosuppression, digestive tract reaction, and cardiac toxicity induced by chemotherapy and did not lead to additional toxicity and side effects. Therefore, it is an anticancer drug which might have good efficacy and low toxicity and worth further investigation.

## Figures and Tables

**Table 1:** Characteristics of included studies.

First author	Year	No. E/C	Age	Stage	Surgery	Intervention	
						Control group	Experimental group
Eisenhauer [16]	2016	40/39	46.73 ± 14.29/45.98 ± 15.78	III-IVa	Yes	CEF (6 cycles) : CTX 600 mg/m^2^, d1, 8; EPI 100 mg/m^2^, d1; 5-FU 500 mg/m^2^, d1, 8; 21d	CT + Aidi 100 ml, qd, d1-8
Chen [17]	2012	30/26	42.5/42.5	II-III	No	AC-T (3 courses) : ADM 60 mg/m^2^, d1+CTX 600 mg/m^2^, d1; 14d, for 4 cycles; followed by TAX 175 mg/m^2^, d1, 14d, 4 cycles	CT + Aidi 100 ml, qd, 4d
Chen [18]	2010	28/20	36.2/37.5	I-IIIa	No	CTF (3 cycles) : CTX 400–600 mg/m^2^, 5-Fu 400–600 mg/m^2^, d1, 8; THP 40–50 mg/m^2^, d1; 21d	CT + Aidi 100 ml, qd, 10d
Fu [19]	2007	44/44	42/48	IV	No	NP (above 2 cycles) : NVB 25 mg/m^2^, d1, 8; DDP 30 mg/m^2^, d2-4; 28d	CT + Aidi 50 ml, qd, d 1–15
Dang [20]	2014	64/64	46.7 ± 20.3	Not clear	Yes	CEF (6 cycles) : CTX 600 mg/m^2^, d1, 8; EPI 100 mg/m^2^, d1; 5-FU 500 mg/m^2^, d1, 8; 21d	CT + Aidi 100 ml, qd, d1-8
Gao [21]	2013a	32/32	33–69	III-IV	No	TAC (at least 2 cycles): TXT 75 mg/m^2^, d1; EPI 90 mg/m^2^, d1; CTX 500 mg/m^2^, d1; 21d	CT + Aidi 80 ml, qd, d1-15
Gao [21]	2013b	16/16	33–69	III-IV	No	GEM + CAPE (at least 2 cycles)：GEM 1 g/m^2^, d1, d8; CAPE 1 g/m^2^, bid, d1-14; 21d	CT + Aidi 80 ml, qd, d1-15
Han [22]	2016	60/60	53.9 ± 11.5/54.2 ± 12.1	Not clear	Yes	CAF/CEF/TAC (6 cycles)	CT + Aidi 100 ml, qd
Jin [23]	2005	50/50	45	II-III	No	CEF (2 cycles): CTX 600 mg/m^2^, d1, 8; EPI 75 mg/m^2^, d1; 5-FUDR 750 mg, d1, 8; 21d	CT + Aidi 100 ml, qd, d1-15
Liu [24]	2006	32/20	46.2 ± 2.6/44.5 ± 3.2	I-IIIa	No	CEF (3 cycles): CTX 400–600 mg/m^2^, 5-FU 400–600 mg/m^2^, d1, 8; EPI 75–90 mg/m^2^, d1; 21d	CT + Aidi 100 ml, qd, 10d
Li [25]	2012	24/28	57.2 ± 3.5/55.7 ± 3.4	IIb-III	No	TAC (2 cycles) : TXT 75 mg/m^2^, d1; EPI 100 mg/m^2^, d1; CTX 600 mg/m^2^, d1; 21d	CT + Aidi 80 ml, qd, d1-15
Lu [26]	2005	60/40	39.5 (20–68）	II-III	No	CMF (3 cycles) : CTX 600 mg/m^2^, d1, 8; MTX 30 mg/m^2^, d1; 5Fu 500 mg/m^2^, d1, 8; 21d	CT + Aidi 80 ml, qd, d1-15
Ren [27]	2013	26/22	42.3/42.2	II-III	No	TC-P (3 courses): THP 60 mg/m^2^, d1, CTX 600 mg/m^2^ d1, 14 d/cycle, for 4 cycles; followed by TAX 175 mg/m^2^, d1, 14d, 4 cycles	CT + Aidi 100 ml qd, 4d
Shen [28]	2006	48/48	51/50	IV	No	CAF or TA (2 cycles): CAF : CTX 600 mg/m^2^, d1, THP 40 mg/m^2^, d1; 5-FU 750 mg, d1, d8; 21d. TA : Taxol 135 mg/m^2^, d1; THP 40 mg/m^2^, d1; 21d	CT + Aidi 50 ml d1-15
Song [29]	2012a	38/41	53.28 ± 11.32/53.02 ± 11.37	Not clear	Yes	CAF/CEF/AC/AT/TAC (6 cycles)	CT + Aidi 50 ml, qd
Song [29]	2012b	32/41	52.67 ± 11.85/53.02 ± 11.37	Not clear	Yes	CAF/CEF/AC/AT/TAC (6 cycles)	CT + Aidi 100 ml, qd
Wang [30]	2016	21/21	46.5 (33–67)	IV	No	NP (at least 2 cycles): NVB 25 mg/m^2^, d1, d8; DDP 30 mg/m^2^, d2, 4; 28d	CT + Aidi 50 ml, qd, d1-15
Wang [31]	2004	31/28	53.5 (31–70)/54.2 (32–69)	IV	No	NT (at least 2 cycles): NVB 25 mg/m^2^, d1, 8; THP 40–50 mg/m^2^, d1; 21	CT + Aidi 50 mg, qd, d1-15; 21d
Yang [32]	2005	30/30	48.4 (31–67)/47.6 (30–65)	III-IV	No	CAF (2 cycles): CTX 600 mg/m^2^, d1, 8; ADM 50 mg/m^2^, d1; 5-FU 500 mg/m^2^, d1, 8; 21d	CT + Aidi 100 ml, qd, d1-10
Yang [33]	2016	36/37	53 (41–67)/54 (38–69)	III-IV	No	TXT + CAPE (6 cycles): TXT 75 mg/m^2^, d1; CAPE 900 mg/m^2^, bid, d1-14; 21d	CT + Aidi 100 ml, d1-14

CT = chemotherapy; E/C = experimental group/control group; 5-FU = 5-fluorouracil; TAX = paclitaxel; NVB = vinnorelbine; DDP = cisplatin; ADM = adriamycin; CTX = cyclophosphamide; EPI = epirubicin; ADM = adriamycin; THP = pirarubicin; TXT = docetaxel; 5-FUDR = floxuridin; GEM = gemcitabine; CAPE = capecitabine.  ^*∗*^Gao Qinghua 2013a were among the treatment-native patients; Gao Qinghua 2013b were among the patients failed in treatment with anthracycline and paclitaxel.

**Table 2:** Summary of comparisons of ADRs among the patients receiving chemotherapy alone and chemotherapy plus Aidi injection.

ADRs	Number of studies	Number of patients	Heterogeneity (*p*/*I*^2^)	Model	OR	*p*
Myelosuppression	7	486	0.80/0%	Fixed	0.34 [0.200, 55]	<0.0001
III-IV myelosuppression	4	259	0.25/28%	Fixed	0.43 [0.25, 0.76]	0.003
Digestive tract reaction	8	623	0.72/0%	Fixed	0.50 [0.33, 0.76]	0.001
III-IV digestive tract reaction	4	269	0.06/59%	Random	0.42 [0.22, 0.80]	0.008
Liver dysfunction	5	432	0.80/0%	Fixed	0.90 [0.48, 1.68]	0.74
III-IV liver dysfunction	1	96	NA	NA	0.48 [0.08, 2.74]	0.41
Leukocyte decrease	3	152	0.41/0%	Fixed	0.43 [0.19, 0.97]	0.04
III-IV leukocyte decrease	4	313	0.03/67%	Random	0.40 [0.24, 0.66]	0.0003
Hair loss	1	88	NA	NA	0.78 [0.30, 2.07]	0.62
III-IV hair loss	1	96	NA	NA	0.71 [0.23, 2.24]	0.56
Phlebitis	2	132	0.61/0%	Fixed	0.89 [0.40, 1.96]	0.77
II-IV cardiac function abnormality	4	320	0.78/0%	Fixed	0.16 [0.13, 0.38]	<0.00001
Atrial dysrhythmia	3	272	0.56/0%	Fixed	0.24 [0.13, 0.45]	<0.00001
Ventricular arrhythmia	3	272	0.96/0%	Fixed	0.08 [0.01, 0.42]	0.003
Atrioventricular block	3	272	0.94/0%	Fixed	0.29 [0.05, 1.80]	0.18
ST segment T wave inversion	3	272	0.91/0%	Fixed	0.25 [0.15, 0.42]	<0.00001
Abnormal ECG	3	272	0.80/0%	Fixed	0.17 [0.06, 0.47]	<0.00001

NA = not applicable; ADRs = adverse drug reactions; OR = odds ratio; ECG = electrocardiogram.

## Data Availability

The data used to support the findings of this study are included within the article.
